# A New Spin on Neural Processing: Quantum Cognition

**DOI:** 10.3389/fnhum.2016.00541

**Published:** 2016-10-26

**Authors:** Carol P. Weingarten, P. Murali Doraiswamy, Matthew P. A. Fisher

**Affiliations:** ^1^Department of Psychiatry and Behavioral Sciences, Duke University Medical CenterDurham, NC, USA; ^2^Brain Imaging and Analysis Center, Duke University Medical CenterDurham, NC, USA; ^3^Duke Institute for Brain Sciences, Duke UniversityDurham, NC, USA; ^4^Department of Physics, University of California, Santa BarbaraSanta Barbara, CA USA

**Keywords:** neurotransmission, neural processing, quantum computing, quantum processing, glutamate, cognition, nuclear spin

Although quantum mechanics is fundamental for understanding molecular mechanisms in physics and chemistry, it has usually been assumed to be unimportant for understanding molecular mechanisms of biological systems. However, there is increasing evidence that quantum mechanics is important for understanding some biological phenomena (Lambert et al., 2013), such as energy transfer in photosynthesis (Fassioli et al., 2014), navigation by birds using the earth's magnetic field (Hiscock et al., 2016), and electron and hydrogen tunneling in biochemical reactions (Klinman and Kohen, 2013). There have also been proposals that quantum mechanics may help explain aspects of brain function.

Discussions about quantum mechanics and the brain began with questions on the role of measurement or observation in quantum mechanics (Stapp, 1991; Theise and Kafatos, 2013). Further developments began to highlight the possibility that quantum mechanics might help explain neural mechanisms involved in consciousness or synaptic function (Stapp, 1991; Beck and Eccles, 1992). Another topic that emerged was whether quantum mechanisms might be employed by the brain to perform calculations, i.e., the possibility of quantum computing in the brain (Penrose, 1989). For example, a model of consciousness was developed that involves quantum computations in neuronal microtubules (Tegmark, 2000; Penrose and Hameroff, 2011; Hameroff and Penrose, 2014a,b; Reimers et al., 2014; Craddock et al., 2015). Other proposals have focused on the quantum phenomenon of spin (see below). Hu and Wu (2004) suggested that nuclear spins of hydrogen, nitrogen, and phosphorus in neuronal cellular components and electron spins of diffusible oxygen and nitric oxide in the brain might mediate consciousness. Electron spins in the brain have also been suggested as a potential target of transcranial magnetic stimulation therapies (Chervyakov et al., 2015). Other perspectives have led to application of quantum probability theory to human decision making (Wang et al., 2014; Kvam et al., 2015). Finally, the above mentioned navigation by birds may involve a quantum mechanical cryptochrome radical-pair (spin dynamic) mechanism in neuronal retinal ganglion cells that transmit information to the brain (Mouritsen et al., 2004; Hiscock et al., 2016).

Recently a new model for how the brain may store and process quantum information has been proposed (Fisher, 2015). The model includes specific biochemical components that could be employed for quantum processing in glutamatergic neurotransmission. It has potential relevance for molecular mechanisms underlying normal neural function, such as glutamatergic dependent neurocognitive systems, as well as psychiatric treatments such as lithium.

## Nuclear spins and quantum processing/computing: neural qubits

This model is based on a quantum phenomenon that underlies something already familiar to neuroscience—magnetic resonance imaging (MRI) (Atlas, 2009). MRI images are made by observing a quantum property of atoms called nuclear spin (Hore, 2011). The most abundant nuclear spin in the brain/body is that of the hydrogen nucleus (^1^H), or proton, that is found in water and numerous other molecules. Most MRI brain imaging is based on observations of proton nuclear spins. Another nuclear spin in the brain is that of phosphorus. Brain imaging of phosphorus nuclear spins has been conducted using magnetic resonance technologies such as magnetic resonance spectroscopy (MRS) and MRS imaging (MRSI). It is phosphorus nuclear spins that are the focus here.

Classical computing is based on information in a binary digit or bit. Quantum processing or computing is based on quantum bits, or qubits, that enable much greater computing power than would be possible using a similar number of classical bits (Bennett and DiVincenzo, 2000; Nielsen and Chuang, 2010). The increase in computing power is the result of quantum phenomena such as superposition and entanglement (Horodecki et al., 2009). Entanglement plays a central role in this model for quantum processing in the brain and more will be said about this below. A variety of nuclear spins can be used as qubits (Vandersypen et al., 2001). Quantum computing with several nuclear spins residing on single molecules that are solvated in water has been realized although it has not been scalable (Nielsen and Chuang, 2010). In the model for quantum processing in the brain, the nuclear spin of phosphorus functions as a qubit, i.e., “neural qubit.”

## Quantum entangeled phosphates

**Figure 1:**
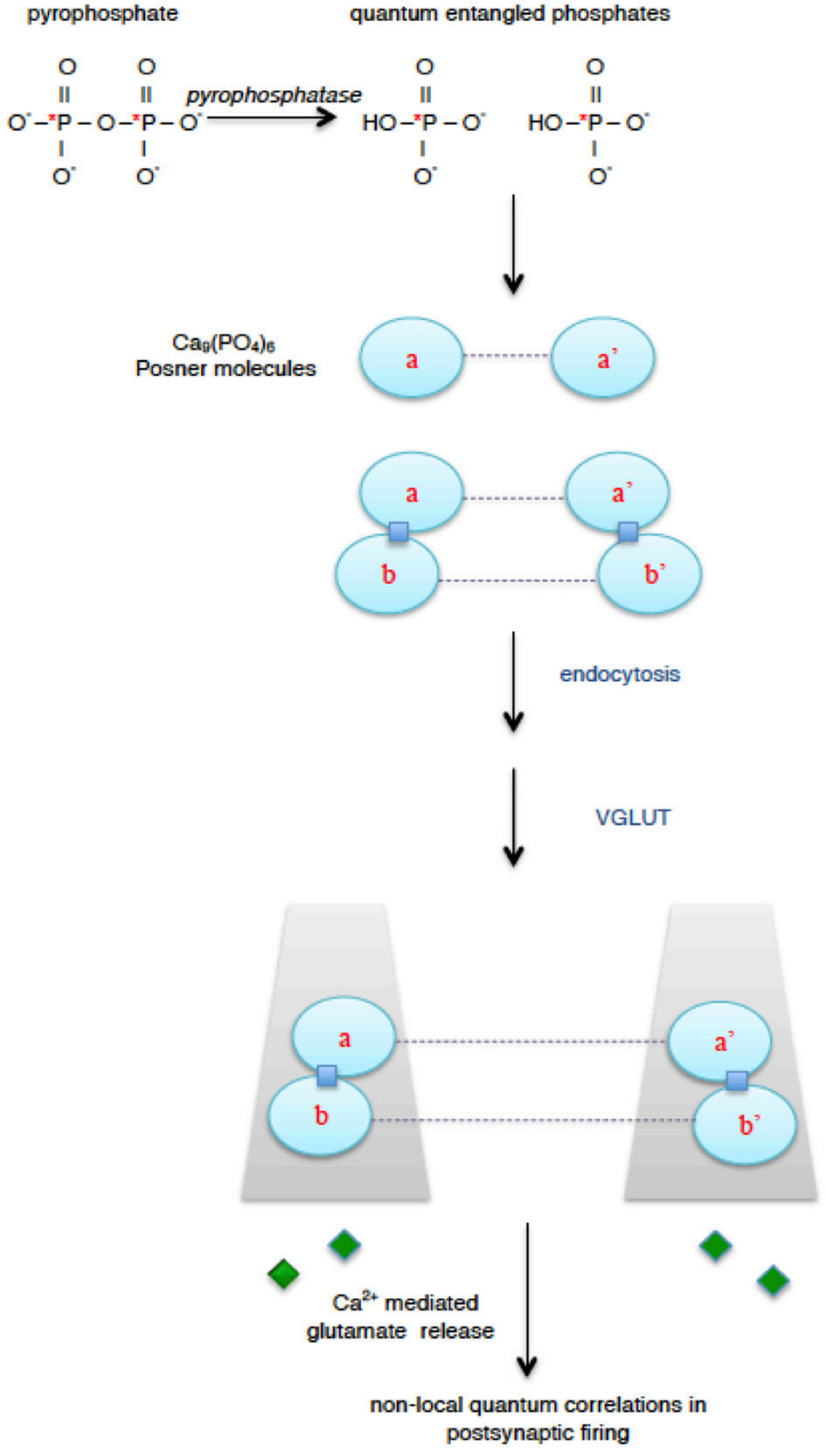
**Proposal for quantum processing in the brain (Fisher, 2015)**. Enzymatic hydrolysis of extracellular pyrophosphate, in which phosphorus atoms can be in a quantum entangled singlet state (^*^P), results in quantum entangled phosphates. The entangled phosphates are incorporated into calcium phosphate Posner molecules producing quantum entangled Posner molecules (dashed line represents entanglement). Two pairs of Posner molecules can undergo binding reactions (squares) to form quantum entangled Posner dimers. Transport of entangled Posner molecules into glutamatergic neurons can be mediated by endocytosis into presynaptic vesicles and action of vesicular glutamate transporter (VGLUT). When entangled Posner molecules in different neurons undergo binding reactions and hydrolysis, this can lead to calcium mediated glutamate (diamond) release from presynaptic neurons and then non-local quantum correlations in postsynaptic firing.

Phosphorus is found in many biological substances including ATP, AMP, inorganic phosphates, bone, creatine, and phospholipids of cell and organelle membranes. The focus here is on inorganic phosphate ${\text{HPO}}_{4}^{2-}$ and pyrophosphate P_2_${\text{O}}_{7}^{4-}$ (Figure 1). Pyrophosphate contains two phosphorus atoms. It is a well-known component of several intracellular and extracellular biochemical reactions, including adenylyl cyclase that converts ATP to the second-messenger cyclic-AMP; uridine diphosphate-glucose pyrophosphorylase; acyl-CoA synthetase; alkaline phosphatase, etc. (Lodish et al., 2000; Terkeltaub, 2001; Yepes et al., 2003).

Pyrophosphate undergoes hydrolysis via enzymatic pyrophosphatases to produce two molecules of phosphate. The phosphate products have an interesting feature at the quantum level: their phosphorus nuclear spins will be predominantly quantum entangled in a very special singlet state (Fisher, 2015). When two spins are entangled in a singlet, a measurement of the state of one of the spins will dictate the result of a measurement on the state of the other spin. This effect is independent of the distance between the two spins. Thus entanglement can lead to a distance independent (non-local) correlation between spins. Although entanglement may sound unusual—Einstein referred to it as “spooky action-at-a-distance”—it is a well observed phenomenon (Gottfried and Yan, 2003; Horodecki et al., 2009; Shalm et al., 2015).

## Posner molecules and quantum processing in glutamatergic neurons

To be useful for neural quantum processing, the entangled phosphates must be transported into different neurons where they can participate in biochemical reactions that are coupled to release of a neurotransmitter such as glutamate. This can result in non-local, quantum correlated postynaptic firing among multiple neurons (Figure 1). The intricate molecular environments of neural intracellular and extracellular spaces mean that important biochemical factors would be needed for quantum processing. A key component is a remarkable calcium phosphate molecule called a Posner molecule Ca_9_(PO_4_)_6._ It was identified as a structural “cluster” in a precursor phase for formation of bone mineral (hydroxyapatite) (Posner and Betts, 1975; Onuma and Ito, 1998; Oyane et al., 1999). Posner's molecules have been observed in simulated body fluids and were approximately spherical with a diameter around 0.87 nm (Dey et al., 2010; Wang et al., 2012). In the model for neural processing, quantum entangled phosphates are incorporated into Posner molecules, and then the quantum entangled Posner molecules are used for quantum memory storage and processing in glutamatergic neurons.

Posner molecules are expected to have several interesting features useful for quantum processing. Firstly, since most naturally occurring isotopes of both calcium and oxygen have no nuclear spin and because of the rapid tumbling expected for the Posner molecules in solvent, the quantum entangled phosphorus nuclear spins are expected to be very protected, remaining coherent for times of a day, or possibly much longer. This would allow Posner molecules to function as a “qubit memory.” Second, chemical binding of two Posner molecules should be a nuclear spin dependent reaction, thereby inducing quantum entanglement between the binding of two separated Posner pairs—this functions as a “measurement” of spin states discussed above. Thirdly, once two bound Posner molecules start rotating about one another their nuclear spin states further entangle. And if/when this rotation stops a further nuclear spin “measurement” is implemented. Once at rest, Posner pairs are more susceptible to “proton attack” and can undergo hydrolysis and “melt” releasing Ca^2+^ into the cytoplasm. This could modulate calcium levels and, therefore, calcium mediated release of glutamate from presynaptic neurons. Finally, because Posner molecules contains six phosphorus atoms they could potentially mediate quantum entanglement and non-local quantum correlations in postsynaptic firing across multiple neurons (Fisher, 2015).

Note that an important step in quantum processing with glutamatergic neurons is the transport of quantum entangled Posner molecules into different neurons (Figure 1). The proposal addressed this and suggested that it could occur through vesicular glutamate transporter (VGLUT). As indicated by its name, VGLUT is the vesicular membrane protein that transports glutamate into presynaptic vesicles (Bellocchio et al., 2000; Takamori et al., 2000; Fremeau et al., 2004; Takamori, 2006). However, VGLUT has also been known as a brain sodium (Na)- dependent inorganic phosphate (Pi) transporter (BNPI) (Werner et al., 1991; Ni et al., 1994, 1996; Bellocchio et al., 2000). In this proposal VGLUT has roles as both a glutamate and phosphate transporter. VGLUT's role as a phosphate transporter is to mediate the movement of quantum entangled Posner's molecules from extracellular space (where they are formed) to the cytoplasm of different presynaptic neurons. This could occur via endocytosis, melting, and reformation of Posner's molecules (Fisher, 2015). The result would be the presence of entangled Posner's molecules in the cytoplasm of multiple presynaptic neurons, which could then lead to post-synaptic firing that is quantum correlated across these neurons.

## Implications for neural processing and neuropsychiatric treatments

Quantum correlations in postsynaptic firing could be naturally involved in a variety of neural systems. These include systems involved in normal neurocognitive processing (thus the term quantum cognition). For example, glutamatergic neurons are neural components of diverse neurocognitive systems throughout the brain. Thus the proposed quantum processing could have a role in neural computation and information processing involved in many types of brain function. These could encompass a variety of normal or abnormal cognitive (including affective and behavioral) functions. They could also be involved in mechanisms underlying treatments of neuropsychiatric disorders (Fisher, 2015). For example, magnetic fields can modulate nuclear spins—the basis for MRI—and an effect on quantum processing might be a mechanism of transcranial magnetic stimulation treatments (Chervyakov et al., 2015). As another example, the mechanisms underlying lithium treatment of bipolar disorder remain obscure. Interestingly, lithium has two isotopes with nuclear spin (^6^Li, ^7^Li), and a remarkable experiment in 1986 found that the two isotopes had opposite effects on the maternal behavior of rats—mothers fed ^7^Li, the predominant isotope in naturally occurring lithium, had suppressed behaviors with low alertness levels, while ^6^Li rats became very active with very high alertness (Sechzer et al., 1986). Quantum chemical calculations have shown that the Posner molecule is stabilized when two lithium atoms replace the central calcium atom (Fisher, 2015). This would alter the phosphorus nuclear spins and modify the quantum neural processing, offering a possible mechanism for the action of lithium (and the difference between the two lithium isotopes).

Perhaps neural quantum processing might also provide a biological architecture that could be “co-opted” and employed for development of laboratory (*in vitro*) quantum computing, similar to how studies of neural circuitry have been contributing to development of artificial intelligence. For example, as indicated by Fisher (2015), a laboratory procedure could be envisioned in which pyrophosphate would be enzymatically hydrolyzed in solution. The nuclear phosphorus spins of the released inorganic phosphates should be predominantly in a spin singlet state and thus quantum entangled (e.g., Figure 1). In the presence of calcium these inorganic phosphates could then form Posner's molecules. Some of these Posner's molecules would be quantum entangled when they incorporated entangled phosphates. The entangled Posner's molecules could then be used in applications of liquid-state nuclear magnetic resonance quantum computing methods (Vandersypen et al., 2001; Oliveira et al., 2007; Nielsen and Chuang, 2010).

## Conclusions

Many studies will be needed to test and further develop this model. Numerous concrete and accessible experiments have been proposed (Fisher, 2015). If this proposal is validated it will radically change our understanding of neural mechanisms involved in normal neurocognitive processing as well as psychiatric disorders and treatments. Our fundamental understanding of the biological basis of the mysterious, and powerful, nature of our cognitive capabilities will now be linked with the mysterious, and powerful, quantum world.

## Author contributions

CW, PD, and MF helped conceive the manuscript. CW drafted the manuscript. CW, PD, and MF made revisions and approved the manuscript.

### Conflict of interest statement

PD has received advisory fees and research grants from several companies. He owns shares in several companies whose products are not discussed here. His work on this manuscript was not supported by any external entity but done on his own time. MF has a U.S. Patent entitled “Treatment for depression and other mental conditions with synthetic isotope-modified lithium” (US 9,044,418 B2). MF had a past research collaboration with Roche. CW declares that her work on this manuscript was conducted in the absence of any commercial or financial relationships that could be construed as a potential conflict of interest.
